# Assessing cancer risk in the anterior part of the prostate using micro-ultrasound: validation of a novel distinct protocol

**DOI:** 10.1007/s00345-023-04591-w

**Published:** 2023-09-15

**Authors:** Sandy Schaer, Arnas Rakauskas, Julien Dagher, Stefano La Rosa, Jake Pensa, Wayne Brisbane, Leonard Marks, Adam Kinnaird, Robert Abouassaly, Eric Klein, Lewis Thomas, Jean-Yves Meuwly, Pamela Parker, Beat Roth, Massimo Valerio

**Affiliations:** 1 Unit of Urology, Department of Surgery, Lausanne University Hospital (CHUV), Rue du Bugnon 46, 1011 Lausanne, Switzerland; 2 Institute of Pathology, Lausanne University Hospital and University of Lausanne, Lausanne, Switzerland; 3 Pathology Unit, Department of Medicine and Technological Innovation, University of Insubria, Varese, Italy; 4 UCLA Institute of Urologic Oncology, Los Angeles, USA; 5 Division of Urology, Department of Surgery, University of Alberta, Edmonton, Canada; 6 Glickman Urological & Kidney Institute, Cleveland Clinic, Cleveland, USA; 7 Unit of Urology, Department of Surgery, Washington University in St-Louis, St-Louis, USA; 8 Department of Radiology, Lausanne University Hospital (CHUC), Lausanne, Switzerland; 9 Department of Radiology, Hull University Teaching Hospitals NHS Trust, Hull, UK; 10 Department of Urology, Geneva University Hospital (HUG), Geneva, Switzerland

**Keywords:** Micro-ultrasound, Prostate biopsy, Prostate cancer, Transitional zone

## Abstract

**Purpose:**

To develop and validate a micro-ultrasound risk score that predicts the likelihood of significant prostate cancer in the anterior zone.

**Methods:**

Patients were enrolled from three expert institutions familiar with micro-ultrasound. The study was conducted in two phases. First, the PRI-MUS anterior score was developed by assessing selected prostate videos from patients who subsequently underwent radical prostatectomy. Second, seven urology readers with varying levels of experience in micro-ultrasound examination evaluated prostate loops according to the PRI-MUS anterior score. Each reader watched the videos and recorded the likelihood of the presence of significant cancer in the anterior part of the prostate in a three-point scale. The coherence among the readers was calculated using the Fleiss kappa and the Cronbach alpha.

**Results:**

A total of 102 selected prostate scans were used to develop the risk assessment for anterior zone cancer in the prostate. The score comprised three categories: likely, equivocal, and unlikely. The median (IQR) sensitivity, specificity, positive predictive value, and negative predictive value for the seven readers were 72% (68–84), 68% (64–84), 75% (72–81), and 73% (71–80), respectively. The mean SD ROC AUC was 0.75 ± 2%, while the Fleiss kappa and the Cronbach alpha were 0.179 and 0.56, respectively.

**Conclusion:**

Micro-ultrasound can detect cancerous lesions in the anterior part of the prostate. When combined with the PRI-MUS protocol to assess the peripheral part, it enables an assessment of the entire prostate gland. Pending external validation, the PRI-MUS anterior score developed in this study might be implemented in clinical practice.

## Introduction

Level 1 evidence shows that early diagnosis of prostate cancer reduces cancer-specific mortality in selected patients [1]. Early diagnosis is achieved through prostate biopsy performed under ultrasound guidance. Recent evidence showed that performing a multiparametric magnetic resonance imaging (mpMRI) prior to biopsy enhances the detection rate of clinically significant prostate cancer as mpMRI-visible lesions can be targeted using modern mpMRI–ultrasound fusion biopsy and has the potential to limit the detection of insignificant disease [2, 3]. However, the inability of conventional ultrasound operating at 8–12 MHz to characterize prostatic tissue is a key limitation, which might be overcome if a real-time tool could be employed to target prostate cancer lesions. Recently, a novel high-resolution transrectal micro-ultrasound (microUS) at 29 MHz has been investigated for prostate cancer detection.

This increased frequency provides superior spatial resolution and allows accurate characterization of the peripheral area of the prostate [4–7]. The study by Ghai et al. validated the Prostate Risk Identification using Micro-Ultrasound (PRI-MUS) protocol with five risk categories, similar to the PI-RADS for mpMRI [5, 8]. Each increase in risk score demonstrated a 10% increase in the probability of clinically significant cancer. In addition, few studies report a potential and independent value of microUS compared to mpMRI [9–11]. Currently, the evaluation of the prostate using the PRI-MUS protocol is only applicable to the posterior/peripheral part of the gland and the ability of microUS to evaluate the anterior area is unknown. The purpose of this study was to develop and validate a microUS risk score to predict the likelihood of significant prostate cancer in the anterior part of the prostate.

## Methods

### Design

This is a multicenter retrospective analysis of consecutive patients undergoing microUS-guided prostate biopsy in three expert centers. This study was approved by the local Research Ethics Committee of each participating institution. MicroUS was performed using the 29 MHz ExactVu transrectal ultrasound system (Exact Imaging™, Markham, Canada). During each session, the prostate gland was scanned from right to left with an imaging depth of 5 cm, followed by an imaging depth of 3 cm. Each scan, as well as targeted and non-targeted biopsies, was saved in a video format using the built-in software. This work was split into two phases: first, a development phase where selected prostate scans in patients undergoing subsequent radical prostatectomy were assessed; second, a validation phase where the previously developed protocol was assessed in a distinct population of patients undergoing biopsy for a clinical and/or biochemical suspicion of significant prostate cancer**.**

### Development of a risk score: anterior part prostate cancer

We selected consecutive patients who had undergone microUS biopsies and radical prostatectomy between May 2019 and June 2022 in the three participating institutions. We included patients with presumed non-metastatic disease at pre-surgery workup, available prostate mpMRI with visual synopsis, available microUS prostate scans and final pathology. For every patient included in this phase, the local expert uro-genital pathologist provided a visual synopsis displaying all prostate cancer foci, the lesion aggressiveness in terms of Gleason Grade Group [12], individual lesion volume and extracapsular extension. This phase aimed to determine the particular microUS features associated with the presence or absence of clinically significant prostate cancer (any Gleason pattern > / = 4) in the anterior part of the prostate which was defined as the area above the prostatic urethra. 102 selected prostate scans were used in the development of the prostate’s anterior zone cancer risk assessment. Investigators initially reviewed both microUS images and final pathology together to describe the appearance of the microUS in areas of the prostate harboring clinically significant prostate cancer and normal tissue. Training material was then produced to describe these differing appearances to new readers.

### Evaluation of the risk score

Seven investigators evaluated the risk scale in an independent set of 50 microUS prostate scans. We selected two groups of patients who underwent both prostate mpMRI and a microUS evaluation in the three participating institutions from May 2018 to October 2020. In the “Anterior Cancer” group, 25 patients with confirmed clinically significant prostate cancer detected in the anterior part of the prostate on either radical prostatectomy or biopsy were selected. In the “Benign Anterior” group, 25 patients with no cancer identified on radical prostatectomy in the anterior prostate were selected; cancers below the level of urethra were permitted. All 50 cases provided full observation of the gland and anterior capsule, with no artifacts significantly obscuring the anterior prostate.

MicroUS prostate scans of the selected patients were shown to investigators with varying level of experience with microUS examination. Each reader was previously trained with the specific module developed above and available online. Every reader assessed the 50 prostate scans and noted for every film whether the presence of significant cancer was likely, equivocal or unlikely in the anterior part of the prostate.

### Statistical analysis

We calculated the sensitivity, specificity and positive and negative predictive values for every reader. Descriptive statistics were used, including the median and interquartile range (IQR) for each metric. To evaluate the coherence between the readers, we calculated the Fleiss kappa and the Cronbach alpha values. Area under the receiver-operator curve (AUC) was assessed for each reader. Statistical analysis was performed with MATLAB (The Mathworks, Natick, MA).

## Results

### Patient characteristics

Median patients’ age was 65 and 67 years, median prostate volume 38 and 40 ml and median PSA 6.6 and 8.6 ng/ml in the development and the validation population, respectively (Table 1).

**Table 1:** Patient characteristics of the development and validation populations

Variable	Development population	Validation population
*N*	102	50
Age, median (IQR)	65 years (59–68)	67
PSA, median (IQR)	6.6 ng/ml (5.2–9.5)	8.6 (6–13)
Prostate volume, median (IQR)	38 ml (28–48)	40 (25–53)
PSA density, median (IQR)	0.19 ng/ml^2^ (0.13–0.28)	0.23 (0.13–0.35)
Final pathology ISUP group, *n* (%)		
1	1 (1)	0
2	42 (41)	10 (40)
3	36 (35)	10 (40)
4	7 (7)	2 (8)
5	16 (16)	3 (12)

### Development of a risk score: anterior part prostate cancer

Initial review of the imaging set produced a large variety of descriptive terms for both benign and cancerous anterior prostate tissue which are listed in Table 2. These terms were then reviewed as a group and simplified into three consensus statements: 1. low-risk anterior prostate tissue has a hyperechoic appearance often with ductal patches and smooth, contiguous capsule; 2. high-risk anterior prostate tissue has a hypoechoic appearance with irregular, poorly defined margins; 3. care should be taken to rule out common artifacts such as edge artifact, shadowing from trapped gas or calcifications, and BPH nodules.

**Table 2:** Association of microUS sonographic features with the presence or absence of clinically significant disease

Feature	Overall (*n*)	Presence of csPCa (*n*)	Absence of csPCa (*n*)	RR	95% CI
Low-risk BPH nodule	32	13	19	0.81	0.49–1.34
Hyperechoic (bright) tissue	28	11	17	0.79	0.46–1.34
Ductal patches	34	12	22	0.71	0.41–1.20
Absence of suspect features	33	10	23	0.61	0.34–1.09
Calcification (shadow) artefact	16	7	9	0.88	0.47–1.63
Shadow artefact	34	11	23	0.65	0.37–1.13
Smooth capsule	28	8	20	0.57	0.30–1.09
Mixed echoes (a mixed echo lesion)	31	20	11	1.29	0.88–1.89
Erased or rubbed charcoal	23	17	6	1.48	1.02–2.14
Storm cloud	35	24	11	1.37	0.96–1.96
Hypoechoic fingers	32	21	11	1.31	0.90–1.91
Smudgy	28	20	8	1.43	0.99–2.05
PRI-MUS apical anterior horn	23	16	7	1.39	0.94–2.05
Ragged borders	35	24	11	1.37	0.96–1.96
Mottled	21	13	8	1.24	0.80–1.91
Distinct lesion	25	17	8	1.36	0.92–2.00

*csPCA* clinically significant prostate cancer, *RR* relative risk

High-risk tissue as described in Table 2 was further grouped by shape and type of irregularity in the margin into four categories: 1. focal lesions with poorly defined, but generally ovular or lenticular margins; 2. lesions with irregular or scalloped margins (“storm cloud” lesions); 3. lesions with irregular finger-like projections; 4. lesions of the anterior apical horn.

**Fig. 1:**
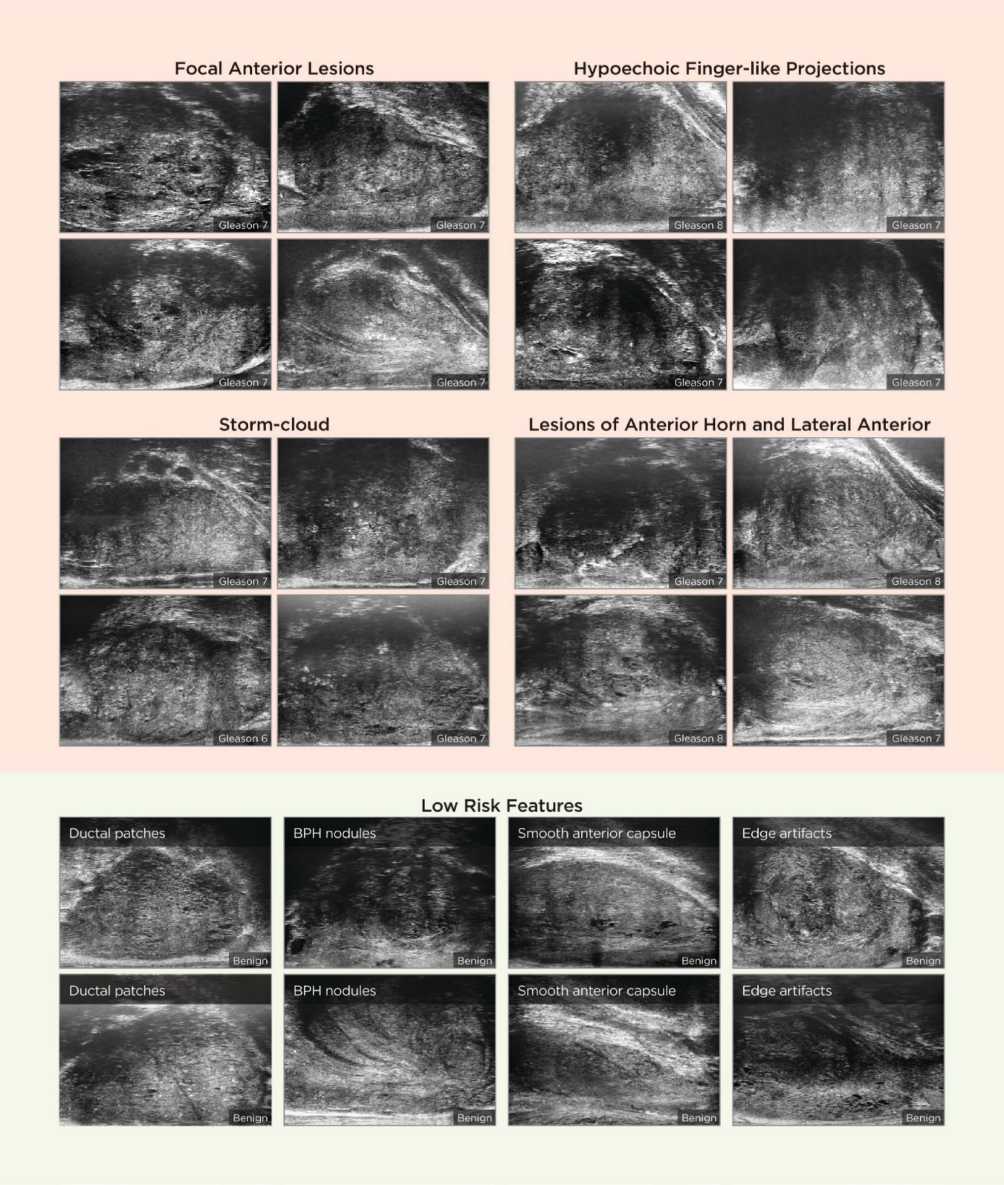
PRI-MUS Anterior score

A novel PRI-MUS anterior score was created using high-risk and low-risk features (Fig. 1).

### Validation of the risk score

**Fig. 2:**
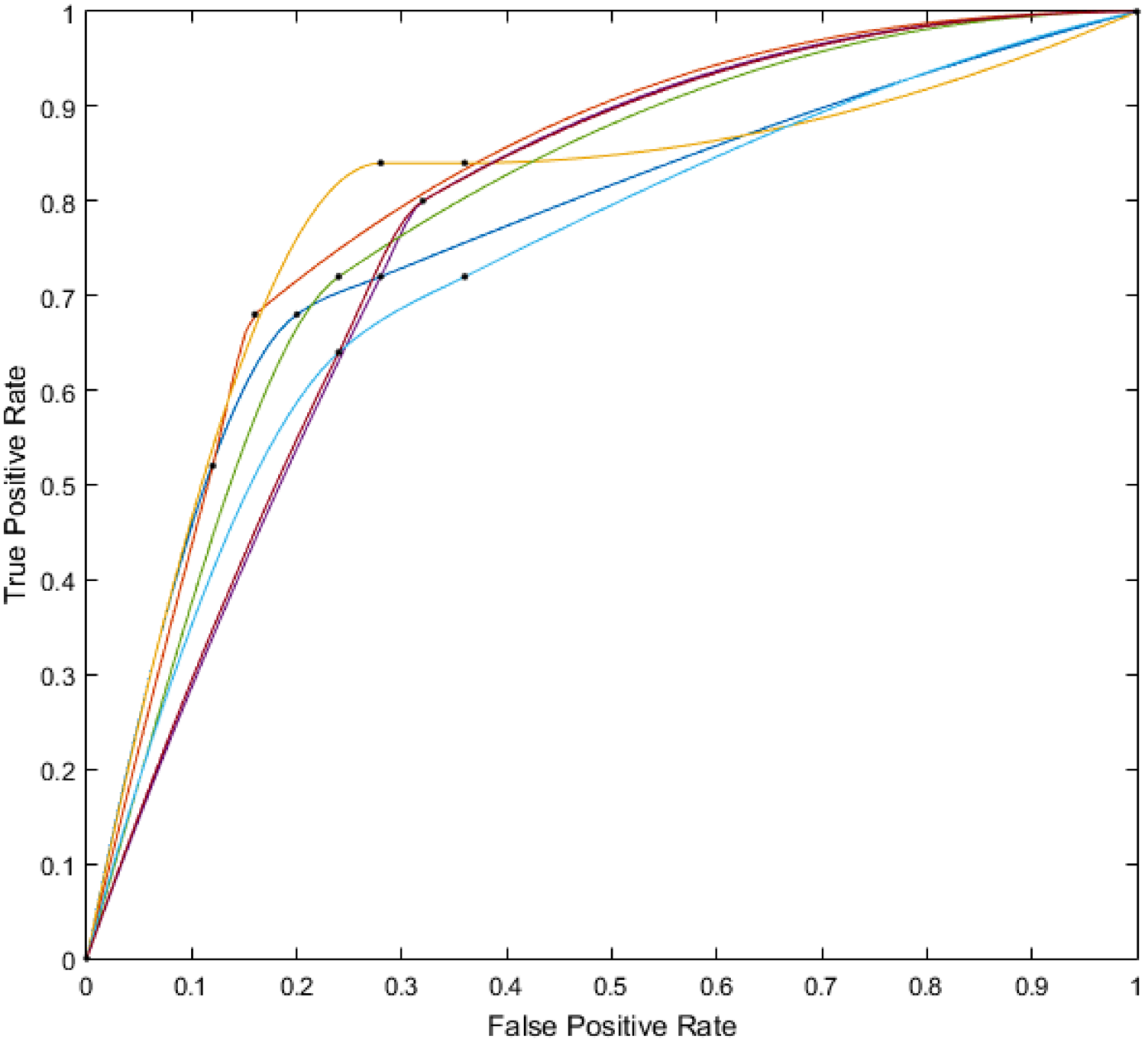
Area under the curve

The median sensitivity, specificity and positive and negative predictive value for all readers were 72% (IQR 68–84), 68% (IQR 64–84), 75% (IQR 72–81) and 73% (IQR 71–80), respectively. If equivocal findings were considered as positive, the sensitivity, specificity and negative and positive predictive values were 72% (IQR 72–80), 68% (IQR 68 –76), 73% (IQR 72–77) and 75% (IQR 75–81), respectively. The accuracy was assessed using the AUC under the individual ROC curves (Fig. 2), with a mean SD of 0.75 ± 0.02 (range 0.71–0.77) The Cronbach alpha value was 0.56, indicating a reliable consistency. Absolute consistency measured by the Fleiss kappa value was 0.179, indicating a slight agreement.

## Discussion

This is the first study suggesting that microUS can be used to characterize prostatic tissue in the anterior part of the prostate. After a short training module, readers with different experience in microUS showed similar diagnostic accuracy. Our study gives a basis to create a standardized protocol to guide microUS users to define the anterior part of the prostate. The suggested PRI-MUS anterior high-risk/low-risk evaluation of the anterior part could be used together with the initial PRI-MUS score developed for the peripheral part to assess the whole gland.

The lack of similar studies makes the comparison challenging. The study by Ghai et al.[5] validated the PRI-MUS protocol for the posterior lesion detection in a similar fashion. The AUC obtained in this study was 0.6 ± 0.02. This value is surprisingly lower compared to our study with an AUC of 0.75 ± 0.02, indicating encouraging diagnostic performance of microUS in the anterior prostate.

We found no study exploring the ability of conventional ultrasound to detect prostate cancer in the anterior part of the prostate. In general, conventional ultrasound has a poor diagnostic accuracy of only 11–35% to define prostate cancer lesions [13]. However, there is emerging evidence on multiparametric ultrasound for whole gland assessment, suggesting that combining ultrasound modalities can improve sensitivity by 13–59% [14]. The CADMUS trial showed that multiparametric ultrasound has a very similar cancer detection rate to mpMRI, detecting only 4.3% fewer cancers than the current standard of care [15]. Nevertheless, this study did not explicitly identify how many of the lesions were in the anterior part. Another recent prospective study evaluated the accuracy of multiparametric ultrasound against radical prostatectomy specimens [16]. In this study, the sensitivity was 67% for transitional zone lesions harboring clinically significant cancer. This is comparable to our results, with a median sensitivity of 72% for clinically significant cancer detection on microUS.

There is growing evidence that microUS might perform equally to mpMRI with a recent systematic review suggesting a similar diagnostic accuracy between the two imaging modalities (detection ratio of microUS against mpMRI 1.05; 95% CI 0.93–1.19) [17]. This is supported by multiple prospective studies [10, 11, 18] highlighting that even with no formal training on recognition of anterior lesions on microUS, the readers were able to detect clinically significant disease as accurately as the current standard of care. Training on recognition of cancer in the anterior prostate should only improve these results. Although the ability of microUS to fully replace mpMRI is yet to be proven, microUS might be considered at present a valuable alternative for those patients who are not eligible to undergo an mpMRI, such as subjects with claustrophobia or with ferromagnetic implants objects not compatible with MR.

The strengths of our study lie in its multicenter design and variable experience of readers for microUS. This highlights the applicability of our suggested PRI-MUS anterior score in everyday clinical practice. There are, however, some limitations of our study. The number of patients and readers included was relatively small. Also, there were few large prostates which are usually more difficult to assess with microUS. However, we would argue that the median prostate size of 40 ml is representative of the general population of subjects undergoing prostate biopsy. Moreover, since 2022, the microUS software allows a depth of 6 cm which should allow a complete visualization of very large glands. Another limitation is that the suggested interpretation of anterior part is based on a retrospective cognitive microUS prostate scans. It is unclear how accurately the prostate would be sampled in real time. Finally, as with any new protocol or modality, the consensus on image interpretation will require further validation. With microUS rapidly gaining popularity worldwide, larger datasets will be available to further optimize the diagnostic performance of anterior zone sampling.

The PRI-MUS anterior score combined with the standard PRI-MUS score allows a standardized whole gland assessment during a microUS prostate biopsy (Fig. 1). Further studies are needed to compare microUS performance directly and prospectively against prostate mpMRI, and to further assess the likelihood of clinically significant disease between the suggested patterns in the PRI-MUS anterior score. While the results from randomized trials comparing microUS to prostate mpMRI are awaited [19], our study will allow microUS users to assess the gland in a standardized way when using microUS as an alternative or in complement to mpMRI.

## Conclusion

MicroUS can detect cancerous lesions in the anterior part of the prostate. The protocol developed in this study suggests that this PRI-MUS score is easy to apply and, combined with the PRI-MUS protocol, the entire prostate can be assessed. External validation of these results against standard of care is warranted prior to the wide uptake of this protocol.

## Abbreviations

microUS: Micro-ultrasound
mpMRI: Multiparametric magnetic resonance imaging
